# Supplementary material for: Elaboration of the Homer1 recognition landscape reveals incomplete divergence of paralogous EVH1 domains
Source: Protein Sci. 2024 Jul 11;33(8):e5094. doi: 10.1002/pro.5094 (PMC11237882; doi:10.1002/pro.5094)
Supplement: Supplementary file 5 — Data S1. Supplemental figures. [file PRO-33-e5094-s004.pdf]

Supplemental Figures:

Elaboration of the Homer1 Recognition Landscape Reveals Incomplete Divergence of Paralogous EVH1 Domains

Authors: *Avinoam Singer[1], Alejandra Ramos[1], Amy E. Keating[1,2,3]*

Affiliations
[1]MIT Department of Biology, Cambridge, Massachusetts, USA
[2]MIT Department of Biological Engineering, Cambridge, Massachusetts, USA
[3]Koch Institute for Integrative Cancer Research, Cambridge, Massachusetts, USA

Correspondence
Amy E. Keating, MIT Department of Biology, 77 Massachusetts Ave., Cambridge, MA 02139, USA.
Email: keating@mit.edu

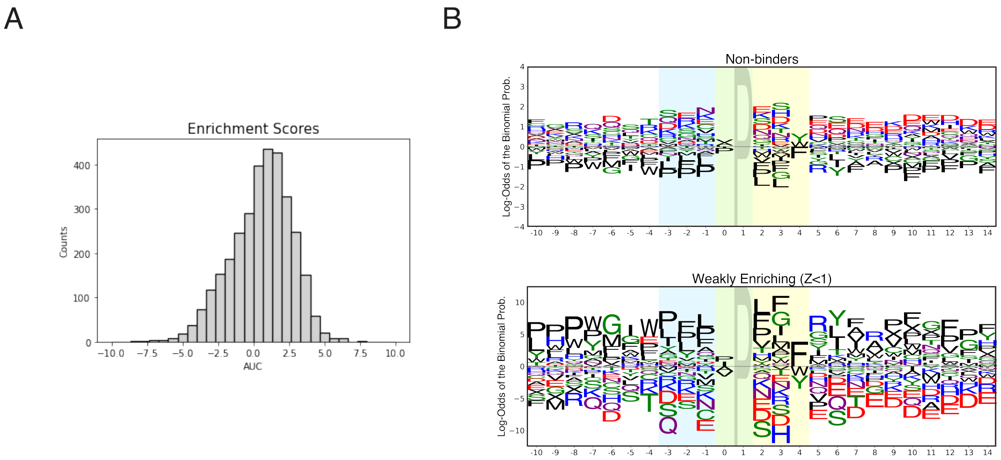

Figure S1: (A) Distribution of enrichment scores for T7-pep peptides. (B) Sequence logos for non-enriching and weakly enriching T7-pep peptides.

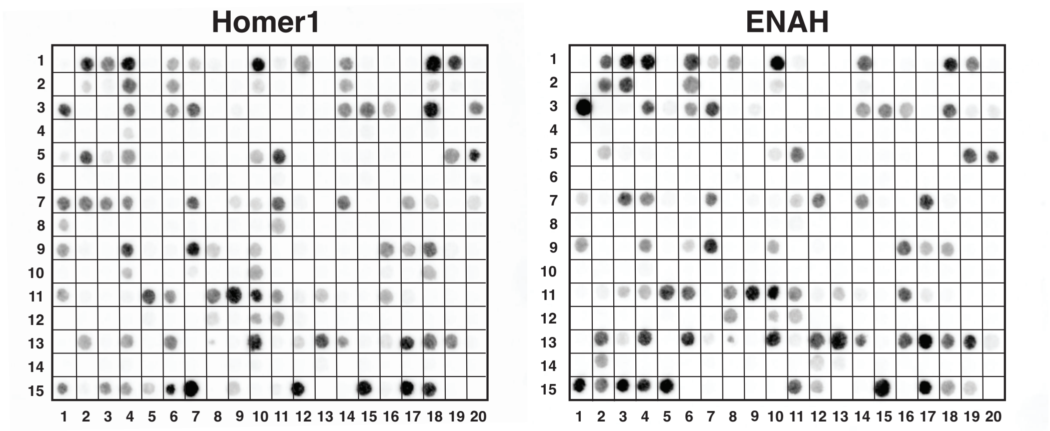

Figure S2: SPOT arrays for Homer1 and ENAH binding to human peptides containing overlapping Ena/VASP and Homer motifs. Corresponding sequences are provided in Supplementary Table 2.

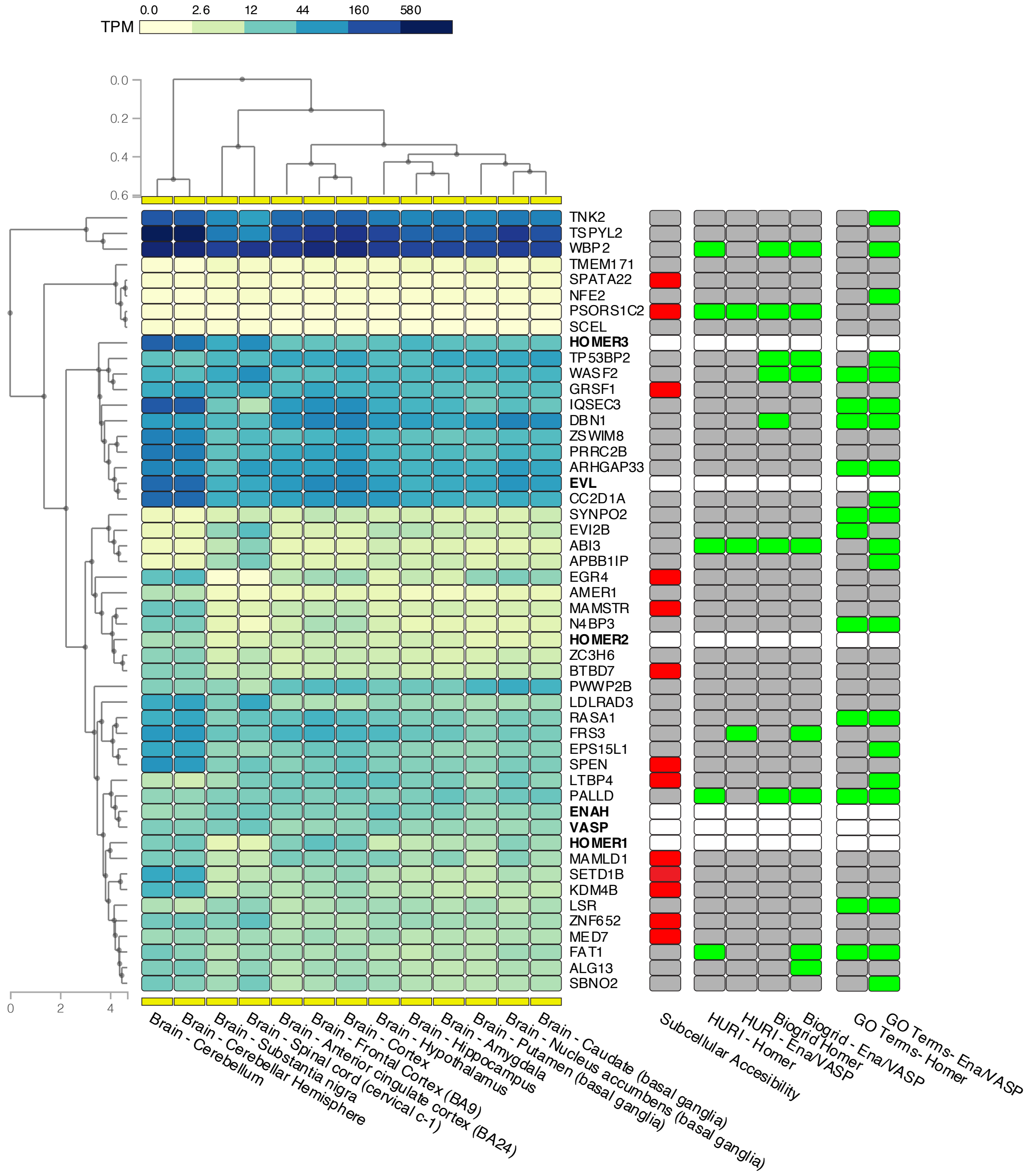

Figure S3: Expression, localization (gTex), and cataloged interactions of proteins containing promiscuous SLiMs (gTex).

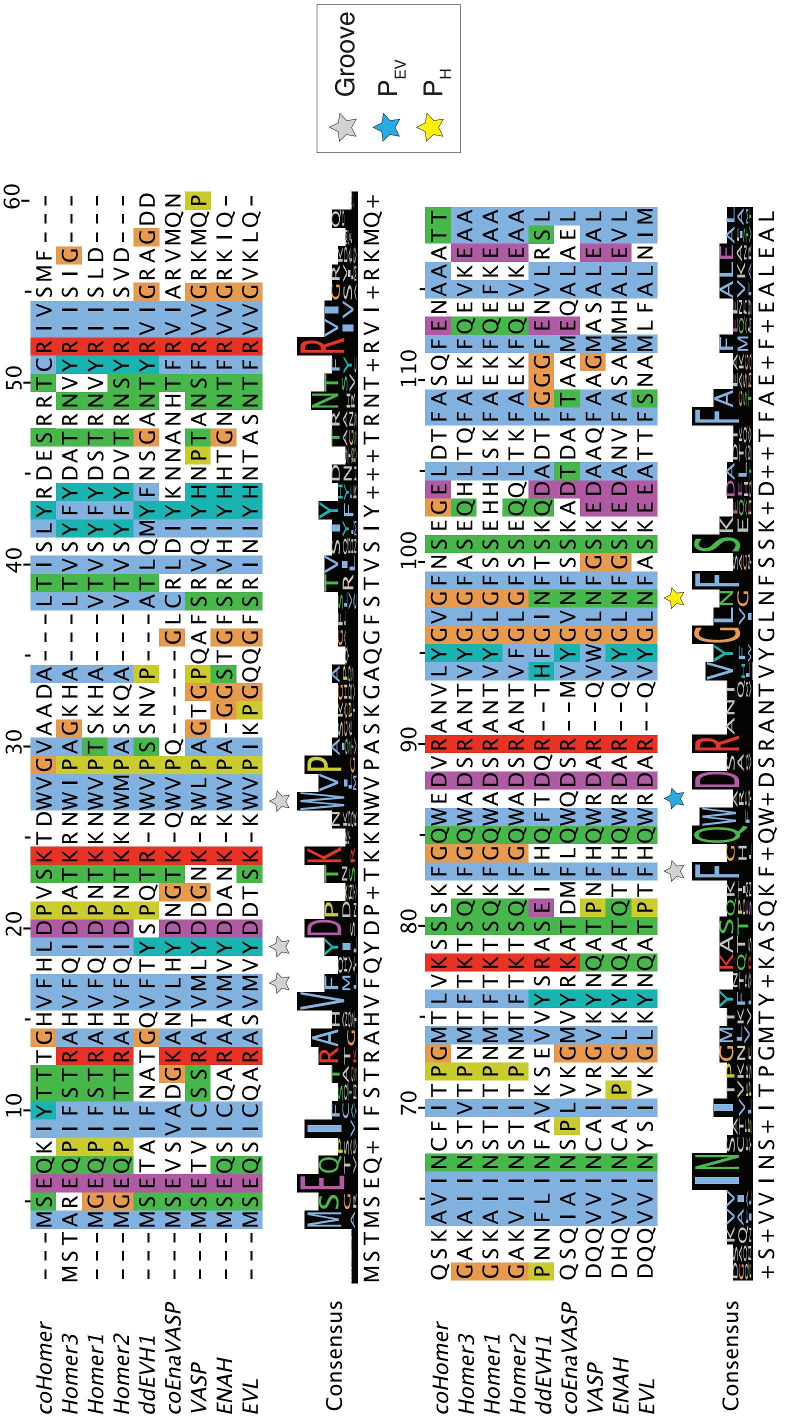

Figure S4: Sequence alignment of the Homer and Ena/VASP EVH1 domains referenced in Figure 6A.

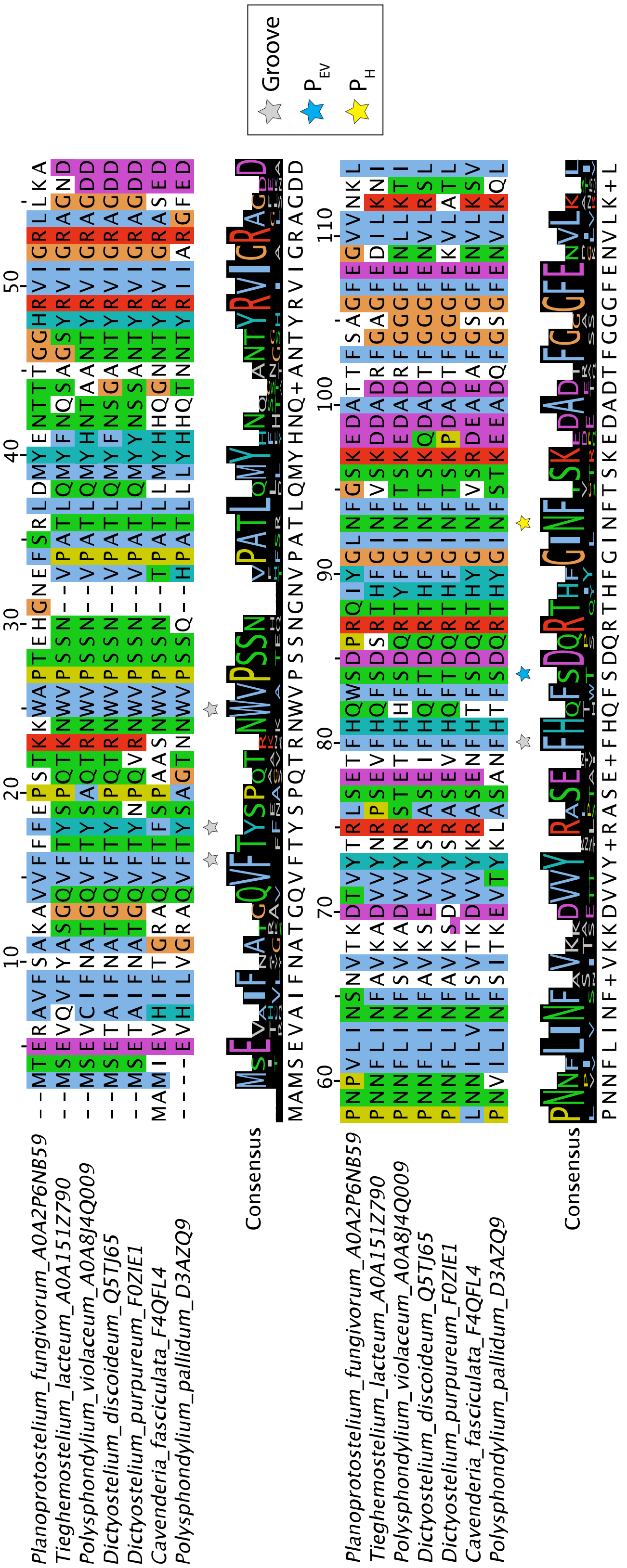

Figure S5: Sequence alignment of Ena/VASP EVH1 domains from amoebas.

A

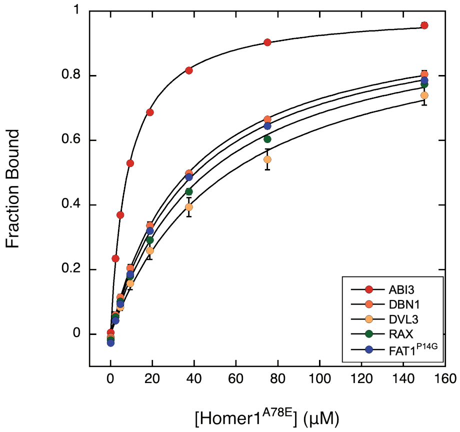

Name	Sequence	K_D (μM)
DBN1	LNFDE**LPEPPATF**CDPEEVE	37.3 ± 1.7
FAT1^{P5G}	DIESD**FPPPPEDF**GAADELP	40.7 ± 1.4
ABI3	GDELG**LPPPPPGF**GPDEPSW	8.2 ± 0.2
RAX	GFGPPAQSLPASYTPPP**PPPPF**LNSPPLGPGLQPLA	46.4 ± 1.4
DVL3	PLPHPGAAPWPMAFPYQ**YPPPPHPY**NPHPGFPELGY	57.4 ± 8.0

B

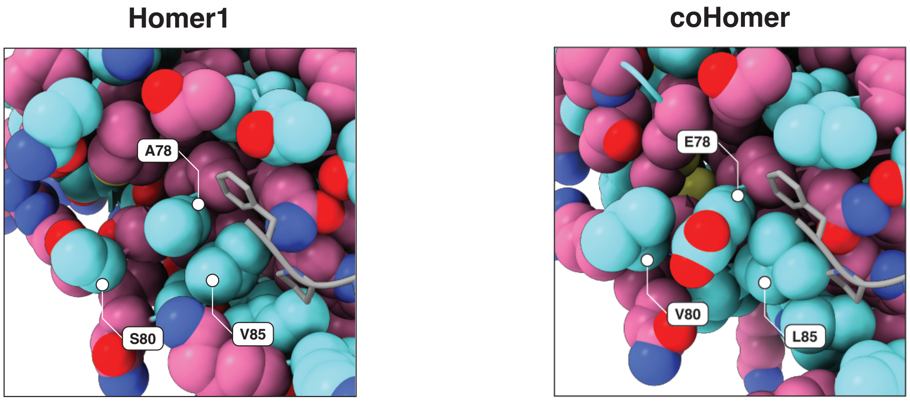

Figure S6: (A) BLI measurements for Homer^{A78E}. Values represent the average and standard deviation of 3 replicates. (B) Comparison of the structural context of E78$_{coHomer}$ (AlphaFold2) and A78$_{Homer1}$ (1DDV).
